# Infective Exacerbation of* Pasteurella multocida*

**DOI:** 10.1155/2016/2648349

**Published:** 2016-01-31

**Authors:** Mayumi Hamada, Noha Elshimy, Hatem Abusriwil

**Affiliations:** Sandwell and West Birmingham Hospitals NHS Trust, West Bromwich B71 4HJ, UK

## Abstract

An 89-year-old lady presented with a one-day history of shortness of breath as well as a cough productive of brown sputum. Her medical history was significant for chronic obstructive pulmonary disease (COPD). She was in severe type one respiratory failure and blood tests revealed markedly raised inflammatory markers; however her chest X-ray was clear. On examination there was bronchial breathing with widespread crepitations and wheeze. She was treated as per an infective exacerbation of COPD. Subsequent blood cultures grew* Pasteurella multocida*, a common commensal in the oropharynx of domesticated animals. The patient was then asked about any contact with animals, after which she revealed she had a dog and was bitten on her left hand the day before admission. We should not forget to enquire about recent history of injuries or animal bites when patients present acutely unwell. She made a complete recovery after treatment with penicillin.

## 1. Background

Many people enjoy the company of pets in their homes, perhaps more so for patients with life-limiting comorbidities and frail elderly patients. This case report highlights some of the dangers of having close contact with animals due to exposure to* Pasteurella multocida* bacteria, which can cause wide ranging manifestations from cellulitis to life-threatening sepsis.

The most important learning point is that we often neglect to ask patients about pets and hobbies, which can be key to the presenting complaint. If these questions had been asked at the initial clerking it is likely that more appropriate treatment could have been instigated earlier. We should also clearly document bruises or bite marks noted on examination as this could trigger our thought process into the mechanism of any underlying injury.

## 2. Case Presentation

An 89-year-old lady presented with a one-day history of acute shortness of breath, as well as a cough productive of brown sputum. Her past medical history was significant for chronic obstructive pulmonary disease, ischaemic heart disease, hypertension, hypothyroidism, and previous breast cancer in 2012, for which she remained on hormonal treatment. She lived with her daughter in a house with a full package of care. However her exercise tolerance was limited to only ten yards with a walking frame, which meant she was confined to downstairs living. She denied ever smoking and drank alcohol within the recommended limits. There was no history within the family of any particular illnesses.

On arrival to accident and emergency she was clearly in respiratory distress. Her observations were as follows: respiratory rate 40, oxygen saturation of 80% on room air, heart rate 130, blood pressure 200/90, and temperature 39.1 degrees Celsius.

On examination there was bronchial breathing with widespread crepitations and wheeze with nil else of significance noted on the initial examination.

An arterial blood gas revealed severe type one respiratory failure and blood tests were indicative of an acute inflammatory response with associated acute kidney injury. This lady was initially treated with intravenous benzylpenicillin and clarithromycin as per trust policy for an exacerbation of chronic obstructive pulmonary disease, as well as being supported with oxygen, intravenous fluids, steroids, and nebulisers. She was acutely unwell and had shown little response after 24 hours. As a result, it was felt that she was unlikely to survive this episode—both the patient and family were informed and the patient was placed on the supportive care pathway with a decision being made not to attempt cardiopulmonary resuscitation and for ward based management only. In the meantime her antibiotics were continued but switched to piperacillin/tazobactam (Tazocin).

She slowly began to respond to treatment and her oxygen requirements were reducing. During the second day of admission the ward received a call from the microbiology consultant stating that the blood cultures grew* Pasteurella multocida* within 24 hours, a common commensal organism in the oropharynx of domesticated animals. This triggered the medical team to enquire about any recent animal contact or injuries. The patient then revealed that she had one dog and two cats at home and had been bitten on her hand by her dog the day previous to admission. This information was new and had not been known previously and it was then noted on examination that there was a healing wound on the dorsum of the left hand. Interestingly the family reported that the dog had been increasingly unwell and aggressive for the previous week, with the vet explaining to the family that the dog had a “brain infection” and needed to be “put down.” Cats are more common carriers of* Pasteurella multocida* than dogs; however she did not provide any history of being recently scratched or licked by her cats [1, 2].

The patient remarkably improved on piperacillin/tazobactam antibiotics, leading to the supportive care pathway being revoked. She was discharged from hospital after receiving ten days of intravenous antibiotics and was sent home with a five-day course of oral coamoxiclav. This lady unfortunately died six months later after a readmission with pleuritic chest pain and acute type one respiratory failure. This was on a background of a recent fall and subsequent fibula fracture. She was too unwell to be investigated extensively but it was felt that the cause of death was likely to be secondary to a pulmonary embolism.

## 3. Investigations

On arrival to accident and emergency an arterial blood gas on 15 litres of oxygen revealed pH 7.448, PaCO_2_ 4.47 kPa, PaO_2_ 9.69 kPa, base excess −0.7, and lactate 3.2. This was in keeping with a significant type 1 respiratory failure.

Blood tests revealed the following: white cell count 38.1 10^9^/L, neutrophils 34.93 10^9^/L, haemoglobin 136 g/L, platelet 280 10^9^/L, sodium 140 mmol/L, potassium 4 mmol/L, urea 6.1 mmol/L, creatinine 114 *μ*mol/L, Glomerular Filtration Rate (eGFR) 40 mL/min/1.7, and C-reactive peptide (CRP) 188 mg/L with normal clotting. These results show clear evidence of an acute infective process with associated acute kidney injury, all in keeping with chest sepsis.

The chest X-ray taken was a portable anteroposterior film. This showed evidence of cardiomegaly and poor inspiratory effort; however the lung fields appeared to be clear.

Blood cultures grew* Pasteurella multocida* within 24 hours of admission (in both aerobic and anaerobic samples). Sputum samples were sent; however this did not grow any organisms.

## 4. Differential Diagnosis

The main differential diagnosis considered in this case was a pulmonary embolism. This was based on the acute deterioration and shortness of breath that she developed within 24 hours and was supported by her relatively normal looking chest X-ray and type 1 respiratory failure. However, this was felt to be less likely as her blood results showed a clear response to an infective focus with significant neutrophilia. Furthermore she was investigated with a Computed Tomography Pulmonary Angiography (CTPA) scan as an outpatient only two weeks before this admission, the results of which were negative.

This led us to treatment as per chest sepsis with an element of exacerbation of her chronic obstructive pulmonary disease. Even though her chest X-ray did not show features of consolidation, clinical examination was very much suggestive of her chest as being the primary focus of infection.

## 5. Treatment

The patient was initiated on intravenous benzylpenicillin and clarithromycin for severe community-acquired pneumonia as per local hospital guidelines. She was also supported with intravenous fluids and oxygen. After 24 hours she remained unwell with little improvement in her inflammatory markers and still requiring significant amount of oxygen to maintain her saturation. Therefore antibiotics were switched to intravenous tazobactam/piperacillin by the medical team. On that same day the results of the blood cultures were revealed. This was discussed with a microbiologist who advised to continue intravenous tazobactam/piperacillin.

## 6. Outcome and Follow-Up

The patient eventually made a full recovery and was discharged home after 10 days of intravenous antibiotics with a short course of oral amoxicillin/clavulanic acid to complete at home.

Unfortunately 6 months later she was readmitted with acute type one respiratory failure on a background of a recent fall with an associated fibula fracture. She was too unwell to be investigated with further imaging and unfortunately died of a suspected pulmonary embolism. Blood cultures on this occasion did not grow any organisms.

## 7. Discussion

*Pasteurella multocida* (*P. multocida*) are small, Gram-negative, nonmotile, facultatively anaerobic, non-spore-forming coccobacilli, measuring 1-2 micrometers in length, which are usually present in the normal flora of the nasopharynx and upper respiratory tract of animals such as cats (70–90%), dogs (50–60%), and pigs (50%) [1, 2]. The mode of infection in most cases is thought to result from direct inoculation of organisms possibly after a bite or scratch injury or from upper respiratory tract colonisation with dissemination to the target organs [3].

Injuries from animal bites are not uncommon and around 250,000 people attend accident and emergency departments in the United Kingdom with animal bites [4]. Most commonly,* P. multocida* infection manifests locally with infections such as cellulitis and local abscess formation [5]. However, it may rarely cause more severe infections such as meningitis, pneumonia, endocarditis, septic arthritis, peritonitis, and empyema, as shown in Table 1 [5]. Furthermore, bacteraemia has been reported in up to 55% of patients with* P. multocida* pneumonia [6].

*Pasteurella* species are associated with a shorter latency period, which is the time from the bite to the appearance of symptoms, compared to staphylococcal or streptococcal infections [8]. In one literature review, 43% of patients developed symptoms of* P. multocida* wound infections within 24 hours after being bitten with rapid onset of intense inflammatory response with local erythema, warmth, swelling, and tenderness [9].

The literature demonstrates that almost all patients with* P. multocida* bacteraemia had chronic underlying diseases and almost all patients who died had severe immunosuppressive disorders [10]. Liver cirrhosis, chronic renal failure, and cancer have all been suggested to be risk factors for invasive* P. multocida* infections [5, 6]. Furthermore, amongst the elderly population affected with pulmonary* P. multocida* infections, the majority had underlying lung disease [11].

In our literature review of* P. multocida* pneumonia cases in the last 10 years, 3 out of 14 cases had chronic obstructive pulmonary disease, as did our case (Table 2). Our review also showed that although most cases had documented exposure to dogs or cats, many of them did not have a history of scratches or bites that were shared by the patients or noted on external examination (Table 2).

The absolute incidence of* P. multocida* infections is unknown as the causative organisms are often not identified in many cases of community-acquired pneumonia and as a result felt to be underreported. In addition, in patients with underlying respiratory tract disease, it is difficult to differentiate between colonisation of the upper respiratory tract and infection.* P. multocida* bacteraemia carries a significant mortality rate shown to be 22.6–23% in two reviews [10, 19].

*P. multocida* is sensitive to most antimicrobial agents: in one review it was found that all* P. multocida* isolates were susceptible to beta-lactams (including penicillin, amoxicillin, and amoxicillin/clavulanic acid), quinolones (including ciprofloxacin), chloramphenicol, tetracycline, and trimethoprim/sulfamethoxazole, whilst 54% were intermediately resistant to aminoglycosides [19]. Another review also highlighted that erythromycin, clindamycin, and 1st-generation cephalosporins (cephalexin, cefaclor, and cefadroxil) should not be used to treat* P. multocida* infections [20].

In conclusion,* P. multocida* can cause life-threatening infections especially in those with serious comorbidities such as chronic obstructive pulmonary disease. Although pet ownership offers psychological support for patients suffering from severe medical conditions, educating patients with such comorbidities about minimising close contact with animals may be a simple solution in preventing life-threatening* P. multocida* infections.

## Learning Points

- Exposure to animals may put frail patients and patients with severe comorbidities at risk of* Pasteurella multocida* infections with a range of clinical presentations from cellulitis to life-threatening sepsis.
- Taking a detailed history of exposure to animals and history of bite injuries/scratches guides clinicians to the causative microorganisms, which guides further management of patients.
- It is important to advise patients with chronic underlying diseases and immunocompromised patients about the dangers of exposure to animals.

## Figures and Tables

**Table 1:** Summary of features of patients with *Pasteurella multocida* septicaemia. This table is a summary of 2 literature reviews on *Pasteurella multocida* septicaemia from 2 large literature reviews based on PubMed search from 1984 to 2003 and 2004 to 2014, respectively [5, 7]. Please note that some patients had more than one underlying disease and presentation of more than one clinical disease.

Patient characteristics	Number of patients
Age	
Infants	28
1–29 years	7
30–59 years	25
>60 years	42
Outcome	
Died	22
Survived	68
Unknown	2
Animal exposure	
Animal exposure	84
No exposure	8
Disease	
Cellulitis	21
Septic arthritis	14
Meningitis	15
Peritonitis	8
Pneumonia	11
Endocarditis	7
No focus of infection	9
Other infections	24
Underlying disease	
Cirrhosis	16
Diabetes mellitus	9
Malignancy	8
Chronic obstructive pulmonary disease	3
Others	30
Healthy	39

**Table 2:** Summary of the findings of a literature review of *Pasteurella multocida *pneumonia in the last 10 years (PubMed search “*Pasteurella multocida *pneumonia”) and *Pasteurella multocida *cases identified in literature review conducted by Christenson et al. [1, 7, 12–18].

Patient characteristics	Number of patients
Age	
Infants	3
1–29 years	0
30–64 years	3
>65 years	8
Outcome	
Died	7
Survived	4
Unknown	2
Animal exposure	
Presence of bites/scratches/licking wound	1
Close contact without bites or scratches	10
No contact	1
Unknown	1
Underlying disease	
Chronic obstructive pulmonary disease	3
Ischaemic heart disease	1
Cirrhosis	2
Malignancy	2
